# Modeling and simulation of the spatial population dynamics of the *Aedes aegypti* mosquito with an insecticide application

**DOI:** 10.1186/s13071-020-04426-2

**Published:** 2020-11-07

**Authors:** Monalisa R. Silva, Pedro H. G. Lugão, Grigori Chapiro

**Affiliations:** 1 Department of Mathematics, UFJF, Juiz de Fora, MG Brazil; 2 Instituto Federal do Sudeste de Minas Gerais, Técnico Panamá 45, Santos Dumont, 36240-000 Brazil; 3 Pós Graduação em Modelagem Computacional-UFJF, Juiz de Fora, 36036-330 Brazil; 4 Pós Graduação em Modelagem Computacional-LNCC, Petrópolis, Brazil

**Keywords:** Spatial Population Dynamics, Modeling, *Aedes aegypti*

## Abstract

**Background:**

The *Aedes aegypti* mosquito is the primary vector for several diseases. Its control requires a better understanding of the mosquitoes’ live cycle, including the spatial dynamics. Several models address this issue. However, they rely on many hard to measure parameters. This work presents a model describing the spatial population dynamics of *Aedes aegypti* mosquitoes using partial differential equations (PDEs) relying on a few parameters.

**Methods:**

We show how to estimate model parameter values from the experimental data found in the literature using concepts from dynamical systems, genetic algorithm optimization and partial differential equations. We show that our model reproduces some analytical formulas relating the carrying capacity coefficient to experimentally measurable quantities as the maximum number of mobile female mosquitoes, the maximum number of eggs, or the maximum number of larvae. As an application of the presented methodology, we replicate one field experiment numerically and investigate the effect of different frequencies in the insecticide application in the urban environment.

**Results:**

The numerical results suggest that the insecticide application has a limited impact on the mosquitoes population and that the optimal application frequency is close to one week.

**Conclusions:**

Models based on partial differential equations provide an efficient tool for simulating mosquitoes’ spatial population dynamics. The reduced model can reproduce such dynamics on a sufficiently large scale.
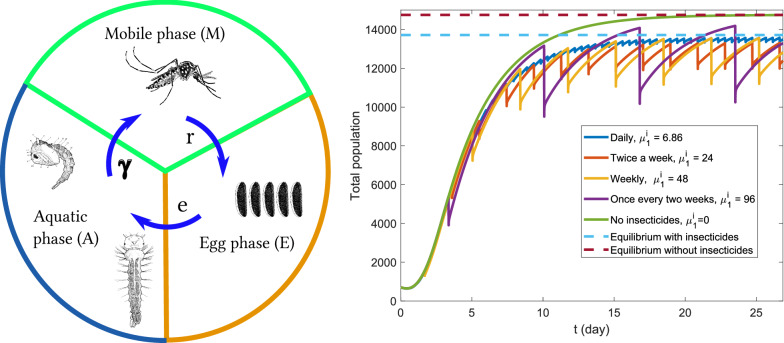

## Introduction

The *Aedes aegypti* (Linnaeus, 1762) mosquito is the main vector that transmits Dengue, Zika, Chikungunya, and Yellow Fever [1]. Urbanization and international travel are key factors that facilitate the spread of these diseases. The study of the spread of mosquitoes and viruses has important implications for understanding diseases, patterns of hyperendemicity, and disease severity, facilitating the planning of public health actions and vaccine development strategies [2]. Dengue is considered among the vector-borne diseases that have spread most rapidly in the world [3]. The Americas, South-East Asia, and Western Pacific are the most affected regions by the dengue fever [4]. Only the Americas region reported 3,139,335 cases through the year of 2019 [5]. Over the past 50 years, this endemic disease grew 30 times, expanding geographically to new countries and, in the current decade, from urban to rural settings [6].

Public policies aiming to control dengue epidemics must necessarily include appropriate strategies for minimizing the mosquito population factor [7]. Some papers address different strategies to control the population of *Ae. aegypti*. For example, using bio-insecticide, larvae-eating fish species, and chemical insecticides [6]; through controlling the breeding of mosquitoes in the home environment during the year [8]; using genetically modified mosquitoes [9, 10]; or in the prospect of sterile insect technique control [11, 12].

There are several approaches to modeling the population dynamics of *Ae. aegypti*. The most common one uses ordinary differential equations (ODEs) following the seminal work by Focks et al. [13, 14]. The importance of temperature and precipitation on mosquito population patterns is investigated in [15, 16]. Authors study the vectorial transmission of diseases using ODEs based on about eight parameters for each spatial location. It is natural to mix this approach with susceptible, infected, and recovered (SIR) models. The authors in [17] use a system of eight ODE equations and approximately fourteen parameters to study the evolution of human infection for Chikungunya of 2005 in several Reunion islands cities.

The modeling approach based on ODEs works with total populations. It can not be used to investigate the spatial dynamics of vectors and related phenomena as terrain topography, different urban areas, etc. For example, some authors [18], circumvent this issue by using a combination of ODEs with the graph theory.

Different possibility to describe the spatial dynamics of the population of *Ae. aegypti* uses partial differential equations (PDEs). This approach is based on the assumption that the vectors’ displacement is an erratic movement and consequently can be modeled as mass diffusion. Several one-dimensional models using this approach have been presented and studied in [7, 19, 20]. However, for these models, it is not possible to analyze the topography of the terrain, considering issues that are very relevant to mosquito propagation, such as heterogeneity and local climatic conditions. A more realistic two-dimensional model can be seen in [21]. However, this complex model considers seven phases of the mosquitoes’ life cycle and results in a significant number (fifteen) of parameters to be determined. In this sense, the current paper follows the work by Yamashita et al. [22], aiming to model the spatial dynamics of the mosquitoes population way, making it possible to model it in a realistic urban scenario. Moreover, we explain how to obtain all used parameters in an attempt to approach mathematical modeling and biological knowledge.

Since estimating all parameters can be challenging, this article focuses on a two-dimensional model depending on few parameters and maintaining the mosquito population dynamics’ main properties, such as the female mobile population and limited carrying capacity of the aquatic phase. We also present how to obtain most of these parameters from experimental data available in the literature using concepts from dynamical systems, genetic algorithm optimization, and partial differential equations. The modeling presented here addresses biological issues and is applied in a real situation considering a heterogeneous scenario in which it is also possible to calculate the population equilibrium. The described approach is used to investigate the impact of the insecticide application frequency in the mosquitoes population.

The paper is organized as follows. "Background" section describes the experiments which form the background of this work. "Methods and modelling" section presents the modeling, explains all parameters and the methods used to estimate them. The numerical algorithm is also described in this section.  "Results" section presents the main results and finally in "Conclusions" section are some discussions and conclusions.

### Background

*Ae. aegypti* and *Ae. albopictus* dispersion in an endemic urban dengue area in southeastern Brazil was analyzed in [23]. They fed adult females on rubidium chloride-enriched blood (RbCl) [24] and measured the dispersal by detecting Rb-labeled eggs in ovitraps. Although there are some limitations in this technique, such as the tip of the proboscises of all rubidium marked mosquitoes were cut off, possibly changing their dispersion [23]. In general, works addressing mosquitoes dispersion distances [25–27] measures the maximum value of such dispersion, making it difficult to evaluate the small distance displacement, which is the norm in mosquito biology [28, 29].

The described experimental results, in our opinion, are more related to diffusion phenomena (connected to the Laplacian operator) then to advection phenomena (modeled as wave propagation). As will be explained in the following sections, it allows us to consider a significant amount of mosquitoes (90% in the current approach) stay in a specific area while the outliers travel further.

**Fig. 1:**
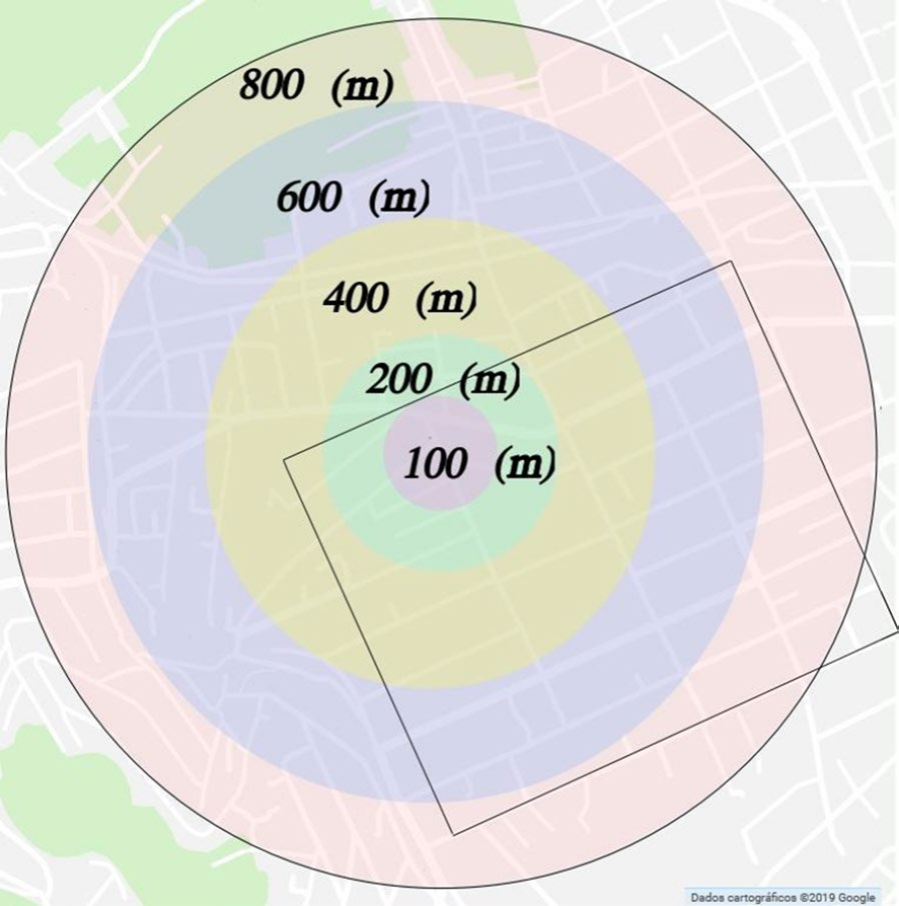
Region used in [23] and its concentric areas, Map of Nova Iguaçu retrieved from Google Maps and highlighted area used in the 2d simulation

The release point at [23] was the center of a circle with a radius of 800 (m). According to [23], two weeks before the release of Rb-tagged mosquitoes, all houses (about 5,000) located in the 1,600 (m) diameter study area were inspected for containers containing immature mosquitoes that were identified and counted. To evenly distribute the ovitraps, the 800 (m) radius circular area was divided into five concentric areas of 0–100 (m) radius (23 ovitraps), 100–200 (m) (69 ovitraps), 200–400 (m) (276), 400–600 (m) (460) and 600–800 (m) (644), respectively, similar to Fig. 1, [23]. In this way, the number of ovitraps per square meter is approximately the same in the investigated area.

Dispersal of *Ae. aegypti*—Fifty-one ovitraps (17 on day 3 and 34 on day 6) were found with Rb-marked *Ae. aegypti* eggs, Table 1. *Ae. aegypti* Rb-marked eggs were found up to 800 (m) from the release point. None of the 23 ovitraps placed up to 100 (m) from the release point was positive for Rb- marked *Ae. aegypti* eggs.

**Table 1:** Number of Rb-tagged eggs recovered in each region [23], the same using analytical solution and numerical simulations, see "Results" section

Distance	Experimental [23]	Analytical	Numerical
0–100 (m)	0	1.56	1.72
100–200 (m)	2	4.52	4.97
200–400 (m)	12	15.77	17.22
400–600 (m)	20	19.63	21.06
600–800 (m)	17	17.74	18.53

## Methods and modelling

### Modelling

**Fig. 2:**
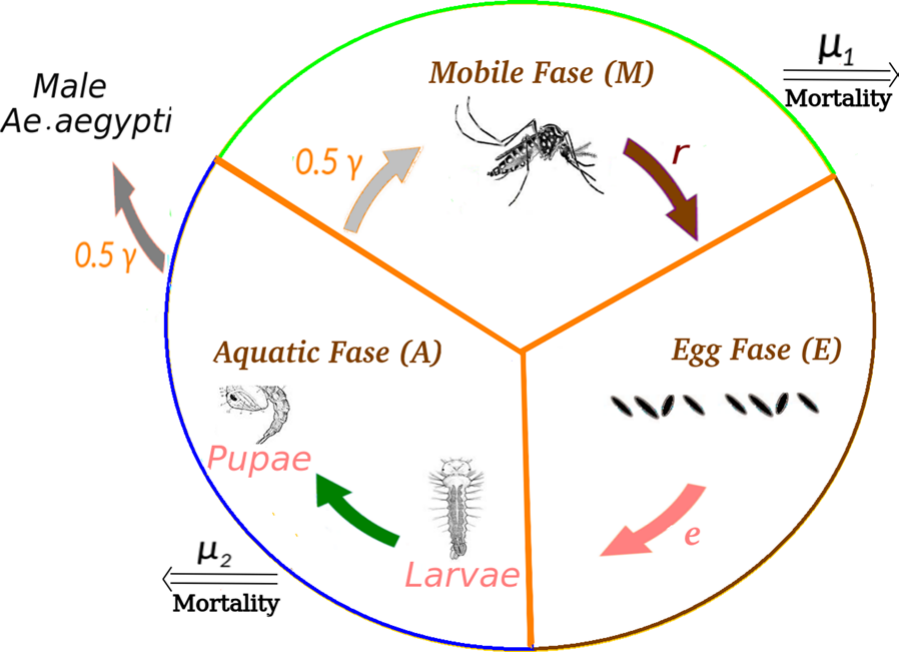
Schematic representation of *Ae. aegypti* Life Cycle. More details are described throughout the  "Methods and modelling" section

We consider four main phases in the life cycle of the *Ae. aegypti*: the mobile female in the reproductive phase (transmits diseases), the egg phase (substantially increases the mosquito population), larva and pupae phases (in this paper we join them into the aquatic phase for simplicity). For simplicity, in this mosquitoes’ population dynamics model, we consider larva and pupae as one phase, see Fig. 2. We are interested in an urban spatial scale, where diffusion represents the dispersion of the mosquitoes due to the autonomous and random movements of the winged females.

Variables *M*, *A*, and *E* represent the population density of *Ae. aegypti* mosquitoes in the mobile, aquatic, and egg phases, respectively. Coefficients $\mu _1$ and $\mu _2$ represent the mortality of the mobile and aquatic phases respectively; *k* is the carrying capacity for the aquatic phase; r represents the oviposition rate of females; D is the diffusion coefficient of females; $\gamma$ is the immobile phase maturation rate; *e* is the hatching rate.

Due to the very high resistance of the egg phase (up to 450 days [30]), as we are interested in an urban spatial macro-scale modeling, we do not consider the mortality in the egg phase. Quantitatively the results presented in this paper do not change significantly considering such parameter. The model is described by the following system of partial differential equations:

1 $$\dfrac{\partial M}{\partial t}= \nabla \cdot (D \nabla M) + \gamma A - \mu _1 M,$$

2 $$\dfrac{\partial E}{\partial t}= r M - e E,$$

3 $$\dfrac{\partial A}{\partial t}= e \left( 1 - \dfrac{A}{k}\right) E - (\mu _2 + \gamma )A,$$

where the domain of variables *M*(*x*, *y*, *t*), *E*(*x*, *y*, *t*), *A*(*x*, *y*, *t*) and initial conditions inside some spatial domain $\Omega \subset {\mathbb {R}}^2$ are given by

4 $$0 \le M(x,y,t) < \infty , \quad M(x,y, 0)= M_0(x,y),$$

5 $$0 \le E(x,y,t) < \infty , \quad E(x,y, 0)= E_0(x,y),$$

6 $$0 \le A(x,y,t) \le k, \quad A(x,y, 0)= A_0(x,y).$$

System (1)–(3) can be regarded as a modification of the model presented in [7], neglecting the term referring convection and dividing the immobile phase into an aquatic phase (larvae and pupae) and an egg phase. This model can also be regarded as a modification of one presented in [21, 31], where we separate eggs from the aquatic phase and consider only the mobile female population.

The carrying capacity, based in [13, 32], represents a space limitation of one phase due to situations present in the environment, such as competition for food among the larvae [33]. The carrying capacity was neglected in the egg phase because of the skip oviposition phenomenon [34]^1^. Limitations in the winged phase were not reported in any study. Finally, we consider the limitation term in the aquatic phase (larvae and pupae), where it is effective [16].

#### Remark

Notice that the variable definition domain given in (6) is invariant under the time evolution by System (1)–(3). In order to prove this affirmative it is sufficient to check that the vector field defined by the right side of (1)–(3) points toward inside the domain, when (*M*, *E*, *A*) approaches the domains border.

1. As the term $\nabla \cdot (D\nabla M)$ can not change the sign of *M*, when *M* is approaching zero, the right side (1) is not negative.
2. The right side of (2) remains positive, when the initial conditions $(M_0,E_0,A_0)(x,y)$ are inside the domains definition, the solution of (1)–(3) remains inside this domain.
3. When *A* approaches zero the right side of (3) is not negative. When *A* approaches *k* (from below) the first term in the right side of (3) tends to zero, while the second term remains negative.

#### How to estimate maturation rate

The time elapsed from the hatching of the *larvae* to the emergency of the *pupae* in the adult phase can be measured experimentally. For example, [35] reports approximately eight days of maturation at a fixed temperature of 26 $^{\circ }$C. To estimate a maturation rate coefficient $\gamma$ from this value, we divide 1 per the maturation time taking into account 50/50 ratio of male/female. The result is $\gamma =0.0625$ female mosquitoes per day.

#### How to estimate oviposition rate

For the oviposition rate, we need to measure the number of eggs per day deposited by a single mosquito. In this case, we use the experimental data from [36], that reports an average of 75.01 eggs per day during five days of the oviposition period at 25 $^{\circ }\text {C}$ with 80% relative humidity. It corresponds to 375.05 eggs in the total lifetime of eleven days seen in the experiment. As the oviposition rate corresponds to an average egg deposition during the mosquitoes’ lifetime, we divide the total number of eggs per lifetime to roughly estimate $r=34$ (eggs/day).

#### How to estimate mortality rates

We assume that all eggs hatch and the corresponding mortality rate coefficient is equal to zero. The mortality rate coefficient of the aquatic phase is defined by the larvae’s coefficient, resulting in the parameter $\mu _2$ approximately equal to 0.025 (1/day) [16].

The mobile phase mortality rate coefficient is calculated as a sum of the base mortality rate and an increment due to insecticides impact: $\mu _1 = \mu _1^{b} + \mu _1^{i}$.

Considering both natural death and accidental ones, approximately $10\%$ of mosquitoes in the adult phase dies at each day [37], giving us a base mortality rate coefficient $\mu _1^{b}$ close to 0.1 (1/day).

In order to model the mortality rate increment due to insecticide impact on the mosquitoes’ population, we add a correction factor to the base mortality rate. To model this factor we consider the Eq. (1) neglecting diffusion term, maturation term, and also neglecting the base mortality rate ($\mu _1 = \mu _1^{i}$):

7 $$\begin{aligned} \frac{\partial M}{\partial t} = - \mu _1^i M. \end{aligned}$$

This type of equation appears in many applications. In particular, for chemical reactions, the characteristic time (time corresponding to complete the major part of the reaction) is defined as $t_{char} = 1/\mu _1$ [38]. We consider the insecticide effect of being 30 minutes, which is the time insecticide suspension stays in the air [39]. Taking this value as a characteristic time, we arrive at the reference value of the mortality rate of $\mu _1^i=48$ (1/day). In what follows, we considered the same application time of 30 minutes for all insecticide application frequencies. The characteristic time for different mortality rates considered in this paper are presented in Table  2.

**Table 2:** Mobile phase mortality rate due to insecticides for different application frequencies and the corresponding characteristic time

Appl. frequency	$\mu _1^i$ (1/day)	$t_{char}$	Duration of effect
Once a day	6.86 by application	3.5 (h)	
Twice a week	24 by application	1 (h)	30 (min) $\approx$ 0.02 (day)
Once a week	48 by application	30 (min)	
Every two weeks	96 by application	15 (min)	

The focus of the model application addressed in this paper is to investigate the impact of the insecticide application frequency in mosquitoes’ population. Thus the total amount of insecticide applied is the same, making it possible to compare different application strategies. For example, if applied every two weeks, the mortality correction factor is $\mu _1^i=96$ (1/day) for thirty minutes; when applied once a week, the mortality correction factor is by $\mu _1^i=48$ (1/day) for thirty minutes and so on.

#### How to estimate diffusion coefficient

While the diffusion coefficient is the most important parameter to define the mosquitoes’ displacement, it cannot be easily estimated from biological aspects as the previous parameters. We use the experimental data [23] to estimate its value through two different approaches: analytical and numerically using the heuristic method. In order to replicate the experiment, the model was simplified: since the focus of the experiment is the dispersal of the initially released Rb-tagged mosquitoes during a short period, the immobile phase equation is neglected. Therefore, the model is simplified to:

8 $$\begin{aligned} \frac{\partial M}{\partial t} = D\nabla ^2M - \mu M, \end{aligned}$$

with the initial condition $M(x,y,0) = M_0\cdot \delta (x,y)$, where $\delta (x,y)$ is a Dirac delta function, and $M_0$ the number of mosquitoes released in the center of the circle. Equation (8) possesses analytical solution for the unbounded two-dimensional domain:

9 $$\begin{aligned} M(x,y,t) = \frac{M_0}{4t\pi D}e^{\frac{-x^2-y^2}{4Dt}- \mu t}. \end{aligned}$$

As this solution decays exponentially with the distance from the origin, considering a sufficiently large domain, the difference from this solution and the correct solution for limited domain on the boundary is negligible. Thus we can use it to estimate mosquitoes distribution.

The **analytical approach** presented in this paragraph is only used to obtain the diffusion coefficient from the experimental data [23]. Notice that the authors in [23] do not apply insecticides and that the total experiment duration was 6 days. During such a short period, the mosquitoes’ mortality does not impact results significantly. Besides, this analytical solution is used to validate the heuristic approach (presented next), which fits diffusion and mortality coefficients. We use the solution (9) without the mortality term ($\mu =0$) and integrate it to find the diffusion coefficient *D*, such that in seven days $90\%$ of the initial population is within the circle of radius 800 (m). While these values seem arbitrary at first, the experiment in [23] shows us that 800 (m) is a minimum radius to consider since mosquitoes can be found in all the explored area. The authors in [40] also corroborate this remark stating that in dry seasons, like the one where the experiment takes place, the mosquitoes can be found at a maximum distance of 1000 (m) from the release point.

For the **heuristic approach**, the experimental data are compared to the simulated one with the following methodology. First, the solution (9) with to-be-fitted values *D*, and $\mu$ is obtained within each of the areas analyzed by the experiment. Each integral value is multiplied by a constant parameter $\alpha$, which indicates the probability of mosquitoes to lay eggs into the ovitraps in the investigated area. The resulting values *R* are compared to the experimental data *E*. A genetic algorithm, described in "Genetic algorithm" section, is used to fit D, $\mu$, and $\alpha$, minimizing the error between *R* and *E*.

#### How to estimate carrying capacity coefficient

The carrying capacity depends on external factors such as food availability, climate factors, terrain properties, making a direct estimation almost impossible. In order to estimate the carrying capacity coefficient *k*, we extend the methodology presented in [13, 41]. Let $\chi \in {\mathbb {R}}^2$ be a part of the domain, where the variables *M*, *A*, and *E* can be considered homogeneous. This assumption agrees with the experimental data, where there is always a limited number of traps. For example, the region $\chi$ can be a block, a neighborhood, or a town.

Considering a compact $\chi$ with smooth boundary $\Gamma$, and assuming a sufficiently smooth solution *M*, Gauss’s Theorem results in:

10 $$\begin{aligned} \displaystyle \iint _{\chi } \nabla \cdot (D \nabla M) dA = \displaystyle \oint _{\Gamma } D\nabla M \cdot \mathbf{n } dS, \end{aligned}$$

where $\mathbf{n }$ is a normal vector pointing outwards the region $\chi$. For simplicity let us consider that $\chi$ is isolated from the neighbor regions. Thus, to estimate carrying capacity coefficient, it was considered that there are no mosquitoes entering or leaving $\chi$ resulting in $\nabla M \cdot \mathbf{n } = 0$ in $\Gamma$.

Under the discussed hypotheses, integrating System (1)–(3) in $\chi$ and dividing the resulting equations by the area of $\chi$, yields the following system of ordinary differential equations:

11 $$\begin{aligned} {\left\{ \begin{array}{ll} \dfrac{\partial }{\partial t} M &{}= (\gamma A - \mu _1 M), \\ \dfrac{\partial }{\partial t} E &{}= (r M - e E), \\ \dfrac{\partial }{\partial t} A &{}= \left( e E \left( 1 - \frac{A}{k}\right) - (\mu _2 + \gamma )A\right) . \end{array}\right. } \end{aligned}$$

Systems similar to (11) were studied in the literature [15, 19, 22, 42]. The solution is the traveling wave connecting two equilibria $(M,E,A)=(0,0,0)$ and $(M,E,A)=(M^*,E^*,A^*)$, where the second one corresponds to the maximum number of mosquitoes. We assume this behavior here as the proof stays outside of the scope of this paper. Equating the right side of System (11) to zero results in:

12 $$\begin{aligned} M^*&= -k\frac{\gamma \mu _1 - \gamma r + \mu _1 \mu _2 }{r \mu _1} = k\frac{\gamma }{\mu _1}\left( 1 - \frac{1}{Q_0}\right) , \end{aligned}$$

13 $$\begin{aligned} E^*&= -k\frac{ \gamma \mu _1 - \gamma r + \mu _1 \mu _2}{\mu _1 e} = k\frac{\gamma r}{\mu _1 e}\left( 1 - \frac{1}{Q_0}\right) , \end{aligned}$$

14 $$\begin{aligned} A^*&= -k\frac{ \gamma \mu _1 - \gamma r + \mu _1 \mu _2}{ \gamma r} = k\left( 1 - \frac{1}{Q_0}\right) , \end{aligned}$$

where:

15 $$\begin{aligned} Q_0 = \frac{r\gamma }{\mu _1\gamma + \mu _1\mu _2} \end{aligned}$$

is equivalent to the basic offspring number [43]. It can be noted that there is a bifurcation here. When $Q_0 \le 1$ the only valid equilibrium inside the variables’ definition domain (6) is (0, 0, 0), since $M^*$, $E^*$ and $A^*$ are non positive. If $Q_0 > 1$, values $0<M^*$, $0<E^*$, and $0<A^*<k$. For more details see [43].

Different experiments obtain the number of eggs, or larvae, or female and male mosquitoes. For example, the authors in [23, 44] collected the number of ovitraps in which the females laid eggs in a determined evaluated region. Another work [45] shows the spatial distribution of *Ae. aegypti* and *Ae. albopictus* larval densities. The authors in [44] investigates the concentration of *Ae. aegypti* females. In this way, using the Eqs. (12)–(14) allows estimating the presented models carrying capacity coefficient for all these cases.

In this work, we obtain a value of the carrying capacity coefficient *k* from the adult mosquitoes estimated population found in [46]. One of its experiments in an urban neighborhood report approximately 100000 mosquitoes in a region of 4000000 ($\hbox {m}^2$), corresponding to $M^* \approx 0.025$ ($\#/\hbox {m}^2$) mosquitoes. The final expression for *k* is computed using this value for $M^*$.

#### How to estimate hatching rate

The experimental data [13] suggests that, in optimal conditions of humidity, the mean value of the hatching rate coefficient is 0.24 (1/day) with a temperature of 28 ($^{\circ }\hbox {C}$), which is considered ideal for the development of the mosquito. In this model, we consider the value $e = 0.24$ (1/day), even though it is known that this parameter is highly dependant on climatic conditions.

### Methods

In this section, we briefly describe numerical methods used in this paper. A simple genetic algorithm is used to fit the parameters by minimizing the error between the experimental data [23] and the simplified model described in "How to estimate diffusion coefficient" section. The finite volume method (FVM) is used to simulate the model (1)–(3).

#### Genetic algorithm

This sections goal is to fit parameter values of *D*, $\mu$ and $\alpha$ by minimizing the error function $\sum ^5_{i=1} ((R_i - E_i)w_i) ^2$. The set of different weights $w_i$ for each region $w = (1,1,5,10,10)$ is used to give more attention to the radial propagation of the mosquitoes. The structure of a genetic algorithm is described in Algorithm 1, see [47] for more details.

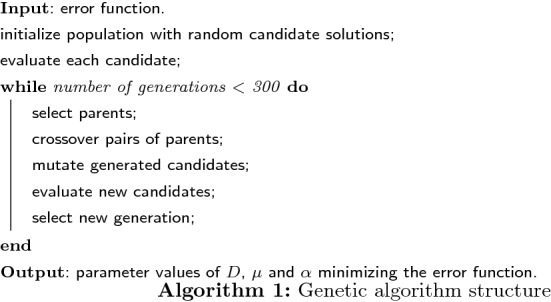



In the Algorithm 1, the selection function, used to choose the best parent candidates and the next generation is given by tournaments of size 4, i.e., between four possible candidates and select the one with minimal error function. The crossover uses an arithmetic function, where the new candidate has the average values between two parents. The solutions are bounded, and the adaptive feasible mutation function ensures that the mutated candidates stay in the defined bounds. Note that the bounds used in the optimization help to achieve a more realistic local minimum of the problem. For the boundaries we consider that the parameters must be positive, D is limited in 30000 ($\hbox {m}^2/$day), $\mu$ is lower than 1 (1/day) and the upper bound for $\alpha$ is estimated to be less than 0.1 because of the considered number of released mosquitoes ($M_0 = 3000$) and the data collected by the experiment.

#### Finite volume method

**Fig. 3:**
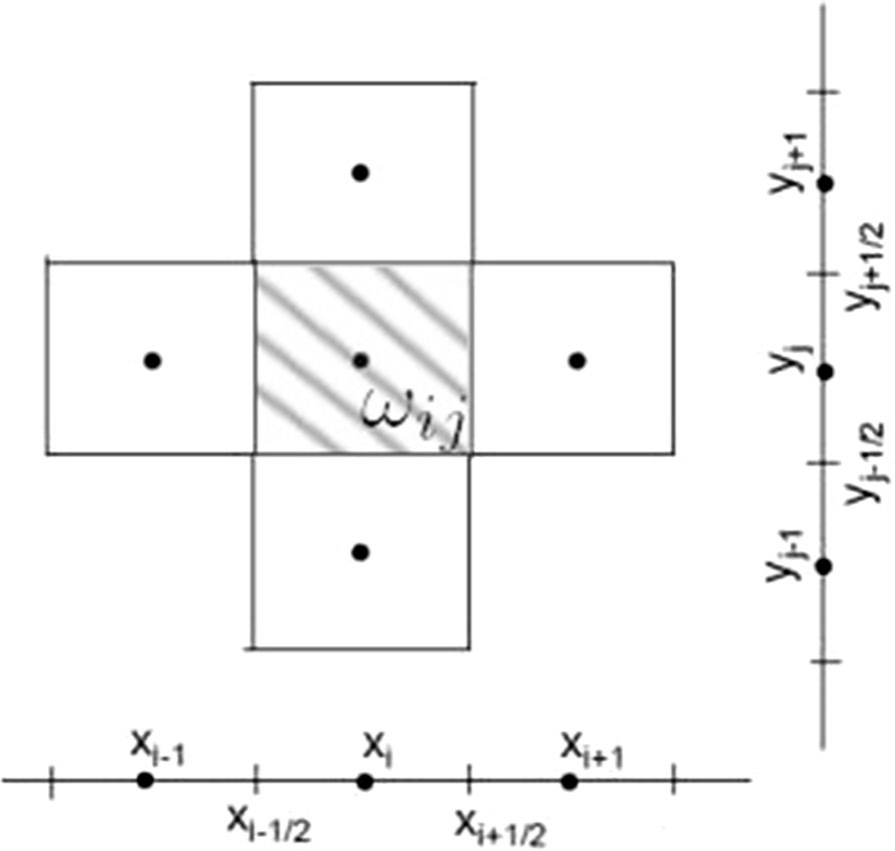
Control volume in finite volume formulation

The governing equations describing the population dynamics of *Ae. aegypti* have been discretized using an explicit FVM [48] detailed next. The domain is given by $\Omega = [0, L] \times [0, L]$. In order to rewrite the System (1)–(3) in the weak form, we integrate it in the control volume $\omega _{ij} \subset \Omega$, see Fig. 3:

16 $$\begin{aligned} \displaystyle \iint _{\omega _{ij}} \frac{\partial M}{\partial t} dx dy= & {} \displaystyle \iint _{\omega _{ij}} \nabla \cdot (D \nabla M) dx dy + \displaystyle \iint _{\omega _{ij}}(\gamma A - \mu _1 M) dx dy, \end{aligned}$$

17 $$\begin{aligned} \displaystyle \iint _{\omega _{ij}}\frac{\partial E}{\partial t} dx dy= & {} \displaystyle \iint _{\omega _{ij}}(r M - eE) dx dy, \end{aligned}$$

18 $$\begin{aligned} \displaystyle \iint _{\omega _{ij}}\frac{\partial A}{\partial t} dx dy= & {} \displaystyle \iint _{\omega _{ij}}\left[ e\left( 1 - \dfrac{A}{k}\right) E - (\mu _2 + \gamma )A\right] dx dy. \end{aligned}$$

Considering $\omega _{ij}$ as a cell centered in $(x_i , y_j )$, we solve each integral separately. For the left side of the System (16)–(18), taking *M*, *E* or *A* as *U*, it follows:

19 $$\begin{aligned} \displaystyle \int _{y_{j-1/2}}^{y_{j + 1/2}} \displaystyle \int _{x_{i-1/2}}^{x_{i + 1/2}} \dfrac{\partial U(x,y,t)}{\partial t} dx dy \approx \Delta x \Delta y \dfrac{U_{i,j}^{n+1} - U_{i,j}^{n}}{\Delta t}, \end{aligned}$$

where $U(x_i,y_j,t_n) = U_{i,j}^n$. We have $\Delta x = x_{i+1} - x_i = y_{j+1} - y_j = \Delta y$ defined by the uniform discretization of the spatial grid, and $\Delta t = t_{n+1} - t_n$ is the time step used in the temporal evolution of the solution.

For the second term in (16) (diffusion term), first consider the derivative only in the X direction:

20 $$\begin{aligned}&\displaystyle \int _{y_{j-1/2}}^{y_{j + 1/2}} \displaystyle \int _{x_{i-1/2}}^{x_{i + 1/2}} \dfrac{\partial }{\partial x} \left( \dfrac{\partial (D M)}{\partial x} \right) dx dy \nonumber \\&\quad \approx \Delta y D\left[ \left( \dfrac{M^n_{i+1,j} - M^n_{i,j} }{\Delta x} \right) - \left( \dfrac{M^n_{i,j} - M^n_{i-1,j} }{\Delta x} \right) \right] . \end{aligned}$$

Using a similar calculation for the Y direction and adding both equations for X and Y directions we obtain the second term in (16) (diffusion term). For simplicity we denote this term $D(M_{ij}^n)$.

The integral of each source term is approximated as follows:

21 $$\begin{aligned}&\displaystyle \int \displaystyle \int _{\omega _{ij}}(\gamma A - \mu _1 M) dx dy \approx (\gamma A^n_{ij} - \mu _1 M^n_{ij}) \Delta x \Delta y, \nonumber \\&\quad \displaystyle \int \displaystyle \int _{\omega _{ij}} \left[ e \left( 1 - \dfrac{A}{k}\right) E - (\mu _2 + \gamma )A \right] dx dy \nonumber \\&\quad \approx \left[ e \left( 1 - \dfrac{A^n_{ij}}{k} \right) E^n_{ij} - (\mu _2 + \gamma )A^n_{ij} \right] \Delta x \Delta y, \nonumber \\&\quad \displaystyle \int \displaystyle \int _{\omega _{ij}}(r M - e E) dx dy \approx (r M^n_{ij} - e E^n_{ij}) \Delta x \Delta y. \end{aligned}$$

Substituting the integrals into (16)–(18) leads to following system:

22 $$\begin{aligned} \dfrac{M_{i,j}^{n+1} - M_{i,j}^{n}}{\Delta t}&=\frac{1}{\Delta x\Delta y}D(M^n_{ij}) +\gamma A^n_{ij}-\mu _{1} M^n_{ij} = F_1(M^n_{ij},A^n_{ij}), \end{aligned}$$

23 $$\begin{aligned} \dfrac{E_{i,j}^{n+1} - E_{i,j}^{n}}{\Delta t}&=r M^n_{ij}-e E^n_{ij} = F_3(M^n_{ij},E^n_{ij}). \end{aligned}$$

24 $$\begin{aligned} \dfrac{A_{i,j}^{n+1} - A_{i,j}^{n}}{\Delta t}&=e\left( 1-\frac{A^n_{ij}}{k}\right) E^n_{ij}-\left( \mu _{2}+\gamma \right) A^n_{ij} = F_2(A^n_{ij},E^n_{ij}), \end{aligned}$$

Using a Crank-Nicolson discretization for the right side of (22)–(24) and rewriting the equations in terms of the previous time and the next time, it follows the implicit scheme:

25 $$\begin{aligned} M^{t+1}_{ij}&= M^{t}_{ij} + \frac{\Delta t}{2}(F_1(M^n_{ij},A^n_{ij}) + F_1(M^{t+1}_{ij},A^{t+1}_{ij})), \end{aligned}$$

26 $$\begin{aligned} E^{t+1}_{ij}&= E^{t}_{ij} + \frac{\Delta t}{2}(F_3(M^n_{ij},E^n_{ij}) + F_3(M^{t+1}_{ij},E^{t+1}_{ij})). \end{aligned}$$

27 $$\begin{aligned} A^{t+1}_{ij}&=A^{t}_{ij} + \frac{\Delta t}{2}(F_2(A^n_{ij},E^n_{ij}) + F_2(A^{t+1}_{ij},E^{t+1}_{ij})), \end{aligned}$$

The simulation consists in solving the nonlinear system (25)–(27) for $M^{n+1}$, $E^{n+1}$ and $A^{n+1}$ at each time step to calculate the population distribution of each phase. We use a time step lower or equal to thirty minutes. More details on this method can be found in [48–50].

#### Simulation of the insecticide application

**Fig. 4:**
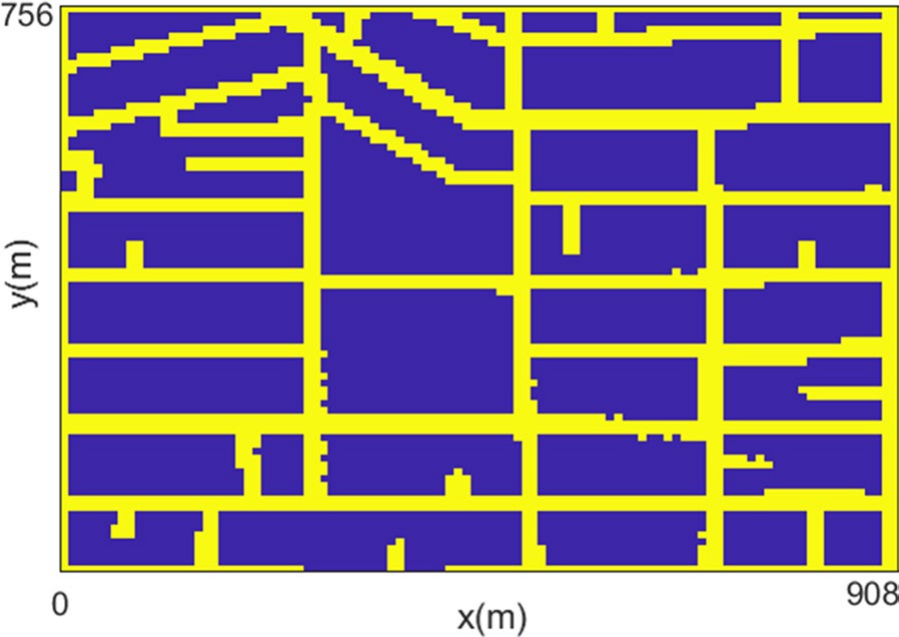
Computational domain corresponding to the area highlighted by a rectangle in Fig. 1. Yellow color indicates the area affected by the insecticide, and blue color indicates the interior of house blocks not affected by insecticide

For the simulations, we consider the area highlighted by a rectangle in Fig. 1. Figure 4 shows the corresponding computational domain, where yellow color indicates the area affected by the insecticide, and blue color indicates the house blocks. Notice that the yellow area is slightly larger than the streets because of the diffusion effect of the insecticide pulverized in the air.

The simulations were performed using Finite Volume Method explained in "Finite volume method" section with initial conditions $M^0_{ij} = 0.001$ ($1/\hbox {m}^2$) and $A^0_{ij} = E^0_{ij} = 0$ ($1/\hbox {m}^2$) for all grid points $(x_i,y_j)$. Parameter values are in Table 5. We run the simulations for two scenarios explained next.

*Heterogeneous scenario* It considers that diffusion coefficient value inside house blocks is equal to half of that obtained in "Parameter estimation" section, since streets are more favorable place for mosquitoes movement. Mortality coefficients inside home blocks are also considered to be 50% of those in streets since there are more natural conditions contributing to mosquitoes’ mortality outside houses, see Table 3. The considered spatial variation of the parameters are hypotheses made by the authors only to show how the model deals with a heterogeneous scenario. Despite being close to fitted values or to values obtained from literature, the exact multipliers corresponding to each city area could not be determined. The average diffusion and average mortality coefficients were maintained equal in heterogeneous and homogeneous scenarios to enable the comparison between both.

**Table 3:** Parameter values used in simulations inside the house blocks and on the streets in the heterogeneous scenario. For the homogeneous scenario we use weighted average of these values

Parameter	Houses	Streets	Homogeneous
*D*	9484.5 ($\hbox {m}^2$/day)	18969 ($\hbox {m}^2$/day)	12440 ($\hbox {m}^2$/day)
$\mu _1$	0.1177 (1/day)	0.2354 (1/day)	0.1544 (1/day)
$\mu _2$	0.0250 (1/day)	0.0500 (1/day)	0.0328 (1/day)

*Homogeneous scenario* It considers that the diffusion and mortality rates of mosquitoes are equal in streets and inside house blocks. Corresponding parameter data for *D*, $\mu _1$ and $\mu _2$ are in Table 3. The importance of this simplified case is that it allows us to make a bridge with the ODE theory, which results are presented in "How to estimate carrying capacity coefficient" section. In order to compare homogeneous and heterogeneous scenarios parameters *D*, $\mu _1$ and $\mu _2$ were calculated as a weighted average between the parameters of streets and houses proportional to the area of the respective environment, see Table 3.

## Results

This section aims to describe the results obtained by the previously described methodologies. "Parameter estimation" section focuses on the parameter estimation using both the genetic algorithm and the analytical approach. "Population dynamics simulation and validation" section shows results comparing the numerical solution to the experimental data and the analytical solution of the model. Simulation results for the complete model with the fitted parameters are also presented.

### Parameter estimation

#### By numerically fitting the experimental data

Given the random nature of the genetic algorithm, it was executed in a 10-fold scheme, calculating the mean value of each parameter, and its standard deviation. The resulting mean and standard deviation are presented in Table 4. The relatively low standard deviation indicates that the results are close to the same local minimum in the limited search space.

**Table 4:** Mean and standard deviations of the parameters from a 100-fold execution

Parameter	Mean	SD (%)
*D* ($\hbox {m}^2/$day)	18969	2660.30 (14%)
$\mu$ (1/day)	0.1177	0.0314 (26%)
$\alpha$	0.0642	0.0140 (21%)

#### By analytical approximation using the heat equation

Due to the experiment’s short duration, Eq. (8) is simplified by removing the mortality term resulting in a heat equation, which possesses a well known analytical solution. Considering initial data given by the Dirac function and using heat kernel [51, p. 45] the two-dimensional solution is given by:

28 $$\begin{aligned} M(x,t) = \frac{M_0}{2\pi \sigma ^2} e^{\frac{-x^2-y^2}{2\sigma ^2}}, \end{aligned}$$

where $\sigma (t,D) = \sqrt{2Dt}$ is the standard deviation, that also represents the “Gaussian width” of the kernel function. We search for the parameter *D*, such that 90% of the initial mosquitoes population stays inside the circle of radius 800 (m) after seven days of the experiment. For all normal distributions, approximately 90% of the area is within 1.64 standard deviations of the mean value, in this case zero. We substitute the values in $1.64\sigma (7,D) = 800$ (m), yielding $D = 16997$ ($\hbox {m}^2/$day).

### Population dynamics simulation and validation

In this subsection, we present the numerical results of the direct simulations using FVM. Initially, we perform a simulation in a 2D homogeneous domain using a simplified model given by Eq. (8). For this simulation, we use parameter values of *D* and $\mu _1$ obtained in previous sections and summarized in Table 5.

**Table 5:** Parameter names and values used in simulations

Parameter	Description	Value	References
*D*	Diffusion coefficient	18969 ($\hbox {m}^2$/day)	Fitted, [23]
$\gamma$	Maturation rate	0.0625 (1/day)	[35]
$\mu _1$	Mobile phase mortality rate	0.1177 (1/day)	Fitted, [23]
$\mu _2$	Immobile phase mortality rate	0.0250 (1/day)	[16]
*r*	Oviposition rate	34 (1/day)	[36]
*e*	Hatching rate	0.2400 (1/day)	[13]
*k*	Carrying capacity	0.0590 (1/$\hbox {m}^2$)	Fitted, [46]

Integrating the numerical solution at $t = 7$ (days) on each of the areas described in Fig. 1 and multiplying the results by the probability of detecting mosquitoes in the trap $\alpha = 0.0642$ gives us values to compare with the experimental data, as seen in the fourth column of Table 1.

The analytical results in Table 1 (third column) are obtained using same parameters in the Eq. (9) and performing the same integration multiplied by $\alpha$.

### Simulation of the insecticide application

**Fig. 5:**
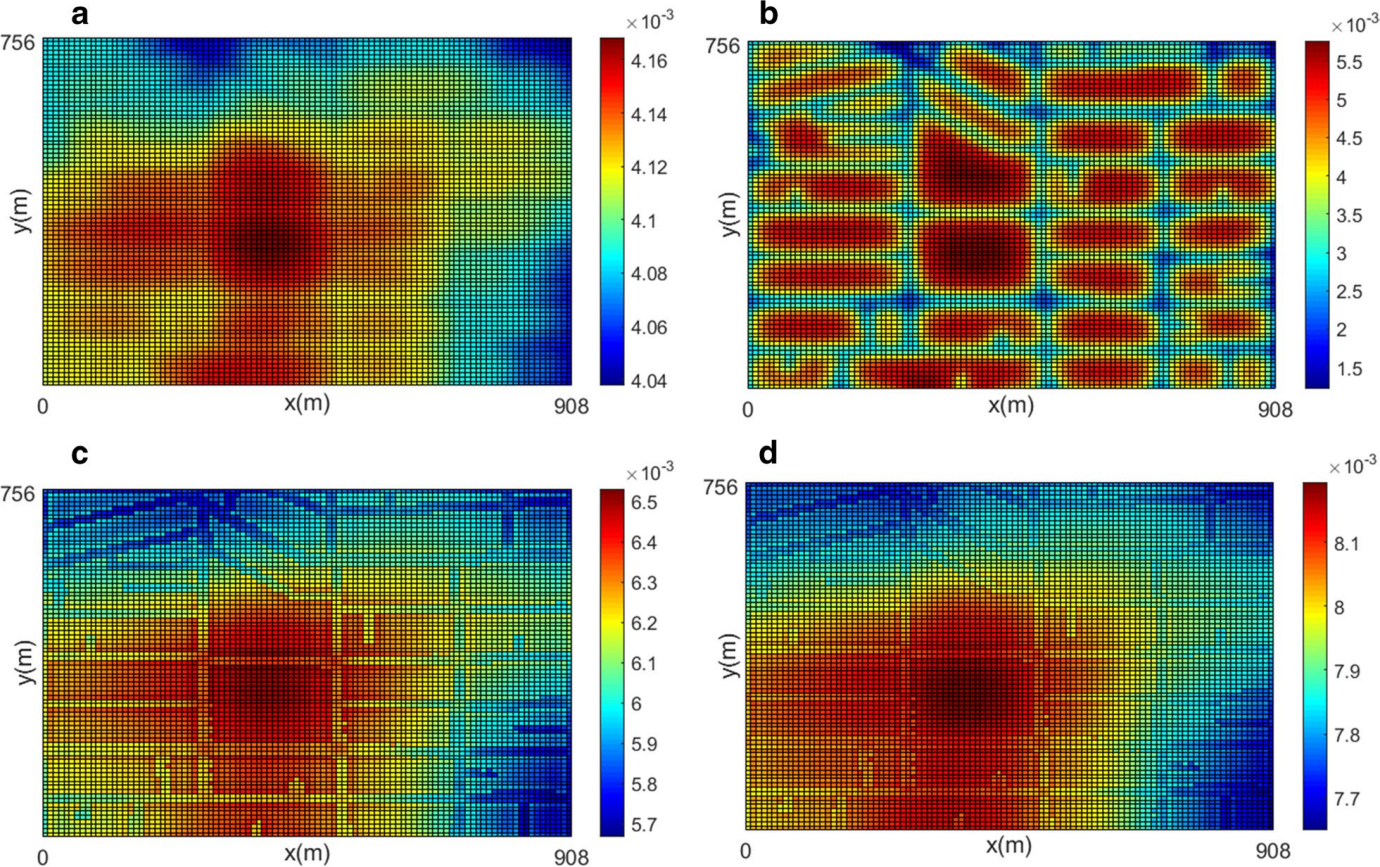
Spatial distribution of mobile phase before the application of insecticides, **a** day 5, during the application, **b** day 6, and after the application, **c** day 7, **d** day 8. Each figure corresponds to the situation at 00:15. The insecticides are applied on day six at 00:00–00:30. The simulation corresponds to the weekly insecticide application

Figure 5 presents mobile phase population density distribution obtained from the simulation of the heterogeneous scenario for weekly insecticide application, see Table 2. Notice that each sub-figure uses its color scale for better understanding.

As can be observed in Fig. 4, there are bigger blocks in the center of the considered neighborhood. Figure 5a shows that, as expected, bigger blocks offer more conditions for the proliferation of the vector. Figure 5b shows that, immediately after the insecticide application, the population density in the streets decreased considerably, while the population inside small blocks is more affected than in bigger blocks. These results are reasonable since pulverized insecticide can not reach areas more distant from the streets. Figures 5c, d show the population recovery after the application of the insecticides. It is clear than the bigger blocks are the source of such recuperation allowed by the presence of the egg and aquatic phases.

**Fig. 6:**
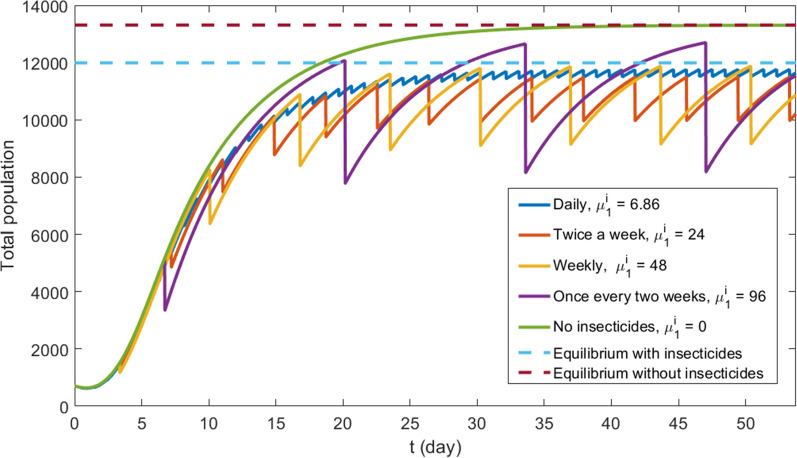
Homogeneous scenario—total population of mobile phase by time with different application strategies. Dotted lines represent equilibria found by Eq. (12)

**Fig. 7:**
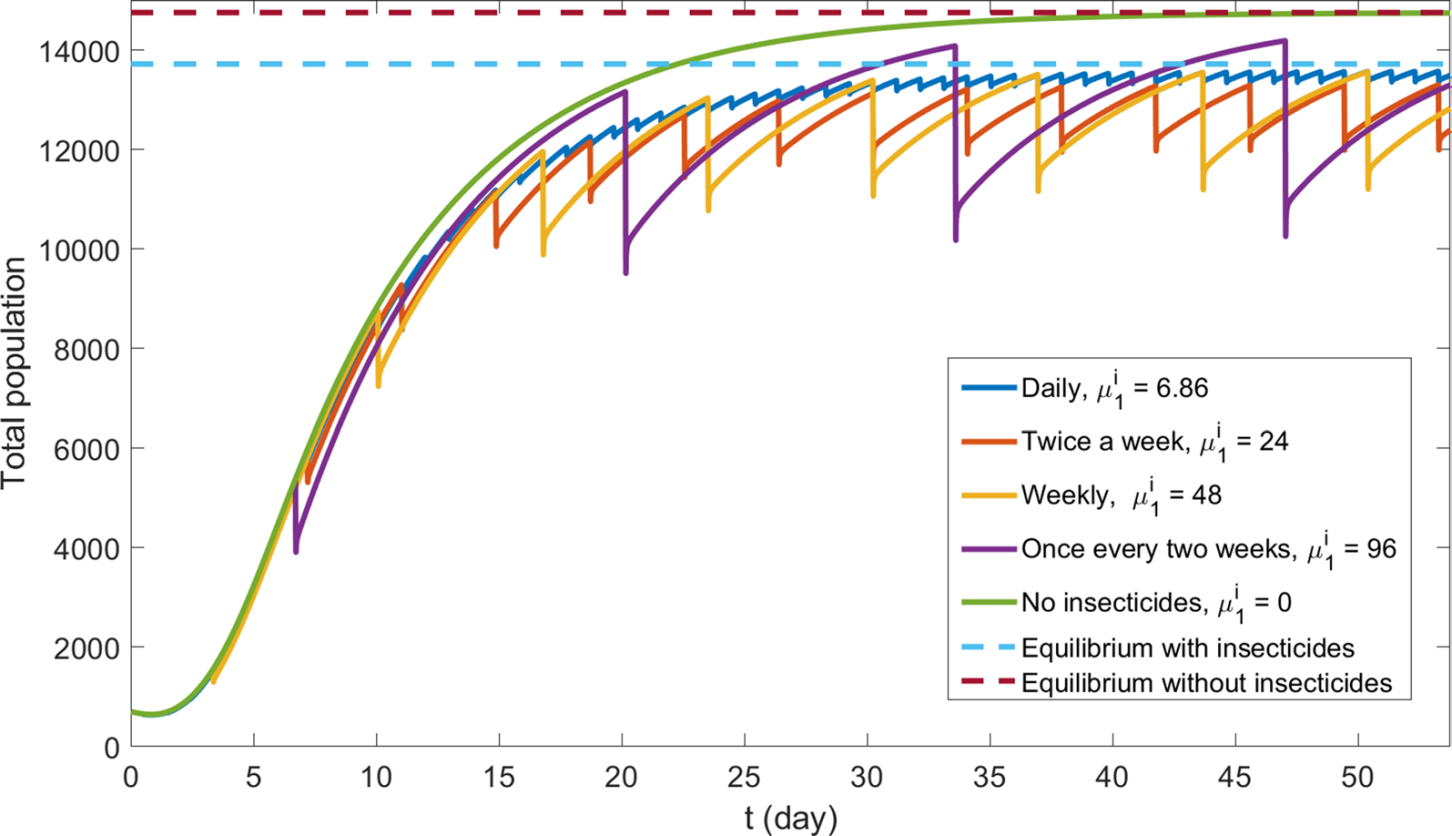
Heterogeneous scenario - Total population of mobile phase by time with different application strategies. Dotted lines represent equilibria found by Eq. (12)

We compare different insecticide application strategies by calculating the integral of the population density in the entire region at each time step. Figures 6 and 7 show total mosquitoes’ population at each day for the homogeneous and the heterogeneous scenarios, respectively. Both figures show five simulations: four simulations with different application strategies (see Table 2) and the case without insecticides use for comparison.

The methodology presented in "How to estimate carrying capacity coefficient" section allows us to calculate an equilibrium state for total mobile mosquitoes’ population using Eq. (12). In the homogeneous scenario without insecticides, we use Eq. (12) directly. To deal with heterogeneous parameters, we apply Eq. (12) to each grid point to find a local equilibrium, and then we sum the results for all grid points to obtain a total population equilibrium. To deal with discontinuous insecticide application, we consider the mortality coefficient (e.g., 48 (1/day)) and divide it by the number of half-hour periods in the corresponding time (in this case 336), simulating the effect of the same insecticide concentration applied continuously. The resulting equilibrium solutions are plotted in Figs. 6 and 7.

As one can see, Formula (12) gives perfect a match for equilibrium solution and no insecticide case in both scenarios. When insecticides are applied, the total mobile mosquitoes’ population oscillates close and below the equilibrium solution in both scenarios. In this case, the total mobile population approximates the equilibrium value given by Eq. (12) when applications become more frequent.

## Discussions

One of the goals aimed by the present article is to evidence the possibility of describing mosquitoes’ spatial population dynamics through a model with few parameters. We presented model simulations using the limited and discrete computational domain using a two-dimensional step function for the spatial variation of the parameters in the heterogeneous scenario. The temporal dependency of parameters is neglected. This simplicity is essential since complex models that rely on a large number of parameters can frequently present limited applications as these parameters are almost impossible to obtain. Numerical fitting of a large number of parameters and limited data inevitably raises the question of a local minimum problem. In our opinion, the spatial dynamics of mosquitoes’ population can be modeled using a diffusion equation.

For the mathematical model cited above, we present how to estimate the main parameter values (diffusion coefficient, mortality rate, and carrying capacity) from the literature [23, 36]. In particular, for the diffusion coefficient, the values obtained through analytical estimates using the heat equation and the numerical fitting through genetic algorithm are close to values found in the literature, evidencing the robustness of the method. We hope the presented methodology will facilitate real applications of these types of models in public health strategies planning.

Equations (12)–(14) allow two applications in the mosquitoes’ population dynamics modeling. Firstly, given experimental data on a maximum number of mobile female mosquitoes or the maximum number of eggs or the maximum number of larvae, they allow the estimate of the carrying capacity of the larvae phase. This coefficient, presented in many models, is almost impossible to estimate otherwise. Secondly, if one knows the carrying capacity coefficient, Eqs. (12)–(14) allow calculating an equilibrium solution for these three phases. Our simulations show that this equilibrium solution is an over-bound for the oscillating mosquitoes’ mobile phase population, even in scenarios when mobility and mortality coefficients are considered different between house blocks and streets. In this case, assuming a direct correlation between the number of mosquitoes and the number of contaminated people, this simple algebraic formula allows us to estimate the effect a given insecticide application strategy will have on public health.

Numerical simulations show that the increased frequency of insecticide application does not imply the decrease of mosquitoes’ population. In fact, more spaced applications lead to bigger oscillations, as can be observed in Figs. 6 and 7. Quantitatively these oscillations are shown in Table 6. Notice that the lower average population corresponds to the weekly application.

**Table 6:** Maximum, minimum and average population of the last 14 simulated days in heterogeneous scenario, corresponding to Fig. 6, for each insecticide application strategy. Between parentheses we show these values relative to the equilibrium solution $M^* = 14752$

Application frequency	Min. value	Max. value	Avg. value
Once a day	13323 ($90.3\%$)	13571 ($92.0\%$)	13472 ($91.3\%$)
Twice a week	11967 ($81.1\%$)	13300 ($90.6\%$)	12818 ($86.9\%$)
Once a week	11189 ($75.9\%$)	13560 ($91.9\%$)	12736 ($86.3\%$)
Once in two weeks	10247 ($69.5\%$)	14187 ($96.2\%$)	13044 ($88.4\%$)

Simulations of the heterogeneous (more realistic) scenario show that mosquitoes’ main population remains inside house blocks and is not accessible to insecticides application. These places work as a source for a fast mosquitoes’ dissemination and population recovery. Taking these results into account together with the damage insecticides cause to other insect species [52] should incentive the debate over the application of this control technique. Better planning optimizes the insecticide application and can diminish such damage.

Finally, it is important to state that more precise results need correct mortality coefficients, which can be obtained through specific experiments.

## Conclusions

In conclusion, our results show the following.

- The simple modeling based on diffusion properties showed satisfactory results for describing the mosquitoes’ spatial population dynamics in the heterogeneous urban environment.
- The total population equilibrium is affected by insecticides’ application, and the periodicity of application plays a significant role in the average total mosquitoes’ population.
- Considering the limitations in data (all parameters are fitted or obtained from the literature) and modeling, our results suggest that the weekly insecticide application results in a local minimum of the average mosquitoes’ population. However, more research needs to be done to determine the optimal strategy for vector control.

## Data Availability

Not applicable.

## Footnotes

1 The female lays the number of eggs that the place holds, without more space, she migrates to other environments to finish laying the eggs.
